# Non-central chirality in organic chemistry

**DOI:** 10.3762/bjoc.22.24

**Published:** 2026-02-24

**Authors:** Ken Tanaka, Naohiko Yoshikai

**Affiliations:** 1 Department of Chemical Science and Engineering, Institute of Science Tokyo, O-okayama, Meguro-ku, Tokyo 152-8550, Japan; 2 Graduate School of Pharmaceutical Sciences, Tohoku University, Aoba-ku, Sendai 980-8578, Japan

**Keywords:** asymmetric synthesis, chiroptical properties, molecular chirality

Chirality is a foundational concept in organic chemistry, traditionally framed around tetrahedral carbon stereocenters. Over the past decades, however, it has become increasingly clear that molecular handedness is not confined to localized points in space. Axes, planes, helices, and more global structural features can equally serve as stereogenic elements, giving rise to what is broadly termed non-central chirality. Rather than constituting a single structural motif, non-central chirality encompasses a diverse family of stereogenic architectures whose commonality lies in how chirality emerges from extended molecular frameworks, dynamic conformational landscapes, or topological constraints. As such, non-central chirality challenges both our synthetic capabilities and our conceptual understanding of stereochemical control.

Numerous reviews have addressed axial, planar, or helical chirality individually, often organized along specific molecular classes or application-driven themes such as asymmetric catalysis or chiral materials. In contrast, this Thematic Issue deliberately brings together diverse manifestations of non-central chirality under a single conceptual umbrella. While admittedly broad, this perspective reflects a growing recognition that these seemingly distinct forms of chirality share common challenges, opportunities, and design principles that transcend traditional classifications.

The contributions collected in this issue illustrate several complementary roles played by non-central chiral molecules in contemporary organic chemistry. First, they continue to serve as demanding benchmarks for asymmetric synthesis, probing how far stereochemical information can be transmitted and controlled when the stereogenic element is spatially or conceptually remote from the reaction site. Second, non-central chiral frameworks remain fertile ground for catalyst design, not only as derivatives of established motifs but also as newly conceived architectures that redefine how chirality can be embedded into catalytic systems. Third, the pronounced chiroptical and functional properties associated with axially, helically, planarly, or inherently chiral molecules underscore their growing relevance in materials-oriented research. Beyond these functional roles, some contributions highlight an even more fundamental aspect of non-central chirality: in certain cases, the very existence of a chiral architecture constitutes a discovery in itself. The identification and stabilization of unconventional stereogenic elements expand the accessible chemical space, prompting us to reconsider what forms of molecular asymmetry are possible, meaningful, and exploitable.

Collectively, the articles in this Thematic Issue should not be viewed as an exhaustive account of non-central chirality but rather as representative snapshots of an evolving landscape. They capture the current state of the field, where structure, reactivity, dynamics, and function increasingly intersect under the shared concept of chirality beyond central chirality. The open and inclusive scope of the *Beilstein Journal of Organic Chemistry* provide a particularly suitable platform for such a concept-driven collection, allowing broad connections across subfields of organic chemistry to be explored without forcing rigid taxonomic boundaries. We hope that this Thematic Issue will encourage readers to look beyond established categories, to recognize common threads among diverse chiral systems, and to further develop non-central chirality as both a practical design principle and a source of conceptual insight.

Ken Tanaka and Naohiko Yoshikai

Tokyo and Sendai, January 2026

## Data Availability

Data sharing is not applicable as no new data was generated or analyzed in this study.

